# An sEMG Denoising Method with Improved Threshold Estimation for Rapid Keystroke Tasks

**DOI:** 10.3390/s26041375

**Published:** 2026-02-22

**Authors:** Pengze Han, Baihui Ding, Penghao Deng, Dengxiong Wu, Huilong Li

**Affiliations:** Key Laboratory of Mechanism Theory and Equipment Design, Ministry of Education, Tianjin University, Tianjin 300350, China; hpz@tju.edu.cn (P.H.); dengpenghao@tju.edu.cn (P.D.); 2023201291@tju.edu.cn (D.W.); long_li1@tju.edu.cn (H.L.)

**Keywords:** surface electromyography, variational mode decomposition, walrus optimizer, improved threshold estimation, noise reduction

## Abstract

**Highlights:**

**What are the main findings?**

- Developed a WO-VMD-based denoising framework for rapid keystroke sEMG, where WO adaptively optimizes key VMD parameters to enhance decomposition stability.
- Proposed an improved threshold estimation for rapid keystroke sEMG to prevent over-suppression induced by thresholding.

**What are the implications of the main findings?**

- Validated on 18 subjects (0–15 dB input SNR), achieving higher ΔSNR and ΔRMSE% than wavelet, EMD, EMD-IT and FCN baselines.
- Provided an “adaptive decomposition + physiologically informed thresholding” approach applicable to rapid keystroke sEMG analysis and other nonstationary biomedical signals.

**Abstract:**

Surface electromyography (sEMG) signals are inevitably affected by noise during acquisition, thereby degrading signal quality and analytical reliability. Most existing denoising methods combine signal decomposition with thresholding, and their performance depends on empirically set decomposition parameters and threshold estimation. However, in high-rate repetitive motions such as rapid keystrokes, sustained high-duty-cycle muscle activation biases universal-threshold noise estimation, leading to unreliable thresholds. To overcome these issues, an sEMG denoising method that integrates the Walrus Optimizer (WO) with Variational Mode Decomposition (VMD) is proposed. WO is employed to optimize key VMD parameters, including the number of modes K and the penalty factor α. Based on this method, an improved threshold estimation strategy is developed to accommodate high-duty-cycle sEMG during rapid keystrokes. It reduces thresholding-induced over-attenuation of meaningful myoelectric components. The dataset included 18 participants with sEMG recorded from six muscles during rapid keystroke tasks (10 trials per participant; 20 keystrokes per trial). Across input signal-to-noise ratios (SNRs) of 0, 5, 10, 15 dB, the proposed method achieved a median SNR improvement (ΔSNR) ranging from 2.75 to 6.65 dB and a median root-mean-square error (RMSE) reduction rate (ΔRMSE%) ranging from 27% to 53%, while maintaining spectral fidelity with a median of median frequency variation rate (ΔMDF%) below 3.48%.These results indicate that the proposed method provides an efficient and reliable solution for sEMG signal processing in rapid keystroke analysis.

## 1. Introduction

sEMG is a noninvasive and easy-to-use technique that has been widely applied in a range of applications, such as clinical diagnosis, prosthesis control, robot control, muscle fatigue detection, and force estimation. However, sEMG signals are commonly corrupted by noise and artifacts, mainly including baseline drift or motion artifacts, white Gaussian noise (WGN), and power-line interference (PLI) [1,2]. Across various applications, the accuracy of sEMG analysis strongly depends on the degree of noise contamination. Consequently, sEMG denoising has become a major research focus in recent years [3,4,5,6,7,8]. Conventional digital filters are the most common and straightforward solution. For example, Waris et al. [9,10] reported mitigating motion artifacts and PLI using a low-pass Butterworth filter and a digital notch filter. However, digital filtering may attenuate informative sEMG components along with noise. This issue is exacerbated when noise and physiologically meaningful sEMG components overlap in frequency. To address these issues, decomposition-based denoising methods have been proposed to improve separability between informative signals and background noise. These methods mainly include wavelet-based denoising, empirical mode decomposition (EMD)-based approaches, and VMD-based methods.

Wavelet-based denoising of sEMG signals is typically performed in three steps: signal decomposition, thresholding of detail coefficients, and signal reconstruction. This procedure leverages the strong time–frequency localization of wavelets, enabling time-varying spectral characteristics in sEMG signals to be represented effectively. Ortolan et al. [11] compared the performance of wavelet methods, adaptive filters, and non-adaptive filters in removing motion artifacts and PLI. Superior performance was observed for the wavelet approach when noise overlapped with the sEMG frequency components. However, wavelet-based denoising requires a priori selection of the mother wavelet, scale, and threshold. These parameters are often chosen empirically, and denoising performance is highly sensitive to their settings [12,13]. Therefore, adaptive data-driven decomposition methods, which generally require less manual parameter tuning, have attracted increasing attention.

EMD is an adaptive data-driven technique that decomposes a signal into multiple intrinsic mode functions (IMFs) and a residual component [14]. EMD-based denoising methods have shown strong potential for nonlinear and nonstationary signals, and relatively few parameters are required. Andrade et al. [3] reported that EMD performed better than wavelet-based techniques for sEMG filtering. Zhang et al. [4] conducted a comparative study on sEMG denoising. They found that EMD-based denoising outperformed conventional filtering. Kopsinis et al. [15] incorporated wavelet-thresholding concepts into EMD-derived modes, thereby improving denoising performance by applying thresholding to noise-dominated IMFs. However, several limitations are associated with EMD, including end effects, mode mixing, and error accumulation from the recursive sifting process. Multiple noise sources are often not fully separated, which can reduce the overall integrity of denoising [16]. Ensemble EMD (EEMD) improves decomposition stability by adding white noise. Mode mixing can be alleviated to some extent, but residual noise may remain in the IMFs [17]. In addition, the frequency resolution of EMD and EEMD is limited by the accuracy of extrema detection. For sEMG signals with spikes and rapid transitions, noise is difficult to suppress effectively within each sub-band [16].

To address these limitations, VMD was introduced by Dragomiretskiy and Zosso [18]. VMD is a non-recursive variational framework for decomposition. In VMD, bandwidth constraints are imposed around each mode’s center frequency, enabling the adaptive extraction of multiple narrowband components from the input signal. This formulation minimizes spectral overlap among the resulting modes and reduces instantaneous frequency fluctuations. Consequently, VMD offers improved robustness in feature extraction and signal denoising tasks [19,20]. Recent studies have further highlighted the strong potential of VMD for sEMG denoising. For example, Xiao et al. [21] combined VMD with wavelet soft thresholding and soft interval thresholding to denoise sEMG signals. Among the evaluated schemes, the combination of VMD and soft interval thresholding yielded the best denoising performance. Ma et al. [22] proposed a VMD-based filtering method to remove multiple noise sources from simulated sEMG signals and reported that the method outperformed conventional filtering methods and EMD. Yous et al. [23] combined a fuzzy-inference-system-based hybrid model with VMD for sEMG denoising, resulting in competitive performance. However, most VMD-based methods still rely on empirical tuning of the mode number *K* and the penalty factor α. Such subjective tuning can destabilize the decomposition and degrade its quality. To reduce the reliance on empirical tuning, metaheuristic optimization provides an effective way to search for suitable VMD parameters under data-driven criteria. Among them, the WO offers strong global search capability with a simple parameterization [24]. Therefore, WO is adopted to automatically determine *K* and α in this study. In addition, many studies estimate thresholds for the decomposed modes using the universal threshold [25]. For sEMG recorded during rapid keystroke tasks, muscle activation is high and sustained. Consequently, the universal threshold tends to overestimate the noise level, leading to overly large thresholds and reduced denoising performance.

In recent years, deep learning has shown promise for denoising in sEMG signal processing. Wang et al. [26] proposed a fully convolutional network (FCN) to remove electrocardiogram (ECG) artifacts from single-channel sEMG when the target muscle is proximal to the heart. Usman et al. [27] trained a one-dimensional convolutional neural network (1D-CNN) to detect and classify various artifacts and contaminants in sEMG signals. TrustEMG-Net is a novel neural-network-based sEMG denoising method proposed by Wang et al. [28], which has demonstrated superior performance on the Ninapro sEMG database compared with existing approaches. Although current deep learning models have demonstrated effective sEMG denoising, training typically requires large annotated sEMG datasets, which limits their use in small-sample scenarios.

To reduce decomposition uncertainty and improve task-adaptive threshold estimation for rapid keystroke sEMG, a denoising method integrating WO-VMD and an improved threshold estimator is proposed. WO is employed to adaptively tune key VMD parameters, thereby reducing reliance on empirical parameter settings. Subsequently, a mode-selection step is performed by evaluating the mean instantaneous frequency of each mode and discarding components outside the physiologically meaningful band. The retained modes are then denoised using the proposed thresholding strategy. Experimental results show improved signal quality in terms of SNR and reconstruction accuracy, supporting the effectiveness of the proposed method for sEMG denoising.

## 2. Methods

**Figure 1:**
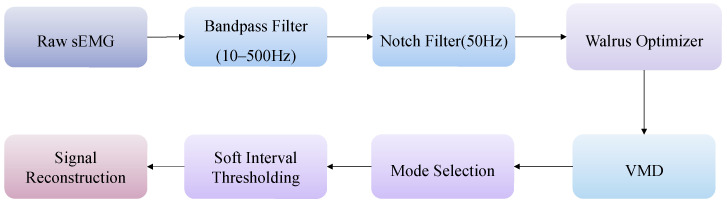
Block diagram for the proposed method for signal denoising.

The overall denoising pipeline is illustrated in Figure 1. The sEMG processing workflow used in this study is described below. Raw sEMG signals were preprocessed using a fourth-order Butterworth bandpass filter (10–500 Hz) and a 50 Hz notch filter to suppress PLI. The preprocessed signals were then fed into WO–VMD, and VMD was performed with the optimal parameter set. The resulting modes were selected, and the selected modes were processed using soft interval thresholding with an improved threshold estimation formula. Finally, the denoised selected modes were summed to reconstruct the denoised sEMG signal.

### 2.1. Variational Mode Decomposition (VMD)

VMD is a non-recursive, variational signal decomposition technique that decomposes an input signal into a set of band-limited modes. Each mode is assumed to be compact around an adaptive center frequency with a limited bandwidth. By casting the decomposition as a constrained variational optimization problem, VMD alleviates mode mixing and boundary effects that are common in recursive decomposition methods. Consequently, VMD yields smooth, narrowband modes and improves the reconstruction fidelity of informative components.

Under the constraint that the sum of all mode components equals the original signal, the bandwidths of individual modes are adaptively optimized, thereby enabling signal decomposition. Accordingly, VMD can be formulated as follows:

(1) $$\underset{\left\{u_{k}\right\},\left\{w_{k}\right\}}{\min}\left\{\sum_{k}{\left\|\partial_{t}\left[\left(\delta (t)+\frac{j}{\pi t}\right)*u_{k}(t)\right]e^{-jw_{k}t}\right\|}_{2}^{2}\right\}$$

(2) $$s.t.\sum_{k}u_{k}=f$$

where f is the original input signal, $\left\{u_{k}\right\},\left\{w_{k}\right\}$ respectively represent the *k*-th mode and its corresponding central frequency, $\partial_{t}$ signifies the partial derivation of a function,δ(t) and ∗ indicate the Dirac function and convolution operator, *j* is the imaginary unit, and “s.t.” means “subject to”, indicating that Equation (2) imposes a constraint on Equation (1).

For solving this constrained variational problem, a quadratic penalty term α and a Lagrange multiplier *λ* are introduced in VMD. The quadratic penalty term is employed to ensure that the sum of all mode components faithfully reconstructs the original signal, whereas the Lagrange multiplier is used to enforce this reconstruction constraint exactly. By incorporating both terms into the objective function, the original constrained variational problem is transformed into the following augmented Lagrangian form:

(3) $$\begin{array}{l} L(\left\{u_{k}\right\},\left\{w_{k}\right\},\lambda )=\alpha \sum_{k}^{K}{\left\|\partial_{t}\left[\left(\delta (t)+\frac{j}{\pi t}\right)*u_{k}(t)\right]e^{-jw_{k}t}\right\|}_{2}^{2}\\ +{\left\|f\left(t\right)-\sum_{k}u_{k}(t)\right\|}_{2}^{2}+\langle \lambda (t),f(t)-\sum_{k}u_{k}(t)\rangle \end{array}$$

Hence, the constrained variational problem is transformed into an optimization problem that seeks the saddle point of the augmented Lagrangian function. The saddle point of the augmented Lagrangian is typically obtained by alternately updating variables using the Alternating Direction Method of Multipliers (ADMMs) [29,30]. The corresponding iterative update rules are summarized as follows:

(4) $$\begin{array}{l} u_{k}^{n+1}=\underset{u_{k}\in X}{\arg\min}\left\{\alpha {\left\|\partial_{t}\left[\left(\delta (t)+\frac{j}{\pi t}\right)*u_{k}(t)\right]e^{-jw_{k}t}\right\|}_{2}^{2}\right.\\ \;\;\;\;\;\;\;+\left.{\left\|f\left(t\right)-\sum_{k}u_{k}(t)+\frac{\lambda (t)}{2}\right\|}_{2}^{2}\right\} \end{array}$$

By exploiting the Parseval/Plancherel isometry property of the Fourier transform, the problem can be solved in the frequency domain:

(5) $${\hat{u}}_{k}^{n+1}(w)=\frac{\hat{f}(w)-\sum_{i\neq k}{\hat{u}}_{i}(w)+\frac{\hat{\lambda }(w)}{2}}{1+2\alpha {\left(w-w_{k}\right)}^{2}}$$

(6) $$w_{k}^{n+1}=\frac{\int_{0}^{\infty }w{\left|{\hat{u}}_{k}(w)\right|}^{2}dw}{\int_{0}^{\infty }{\left|{\hat{u}}_{k}(w)\right|}^{2}dw}$$

(7) $${\hat{\lambda }}^{n+1}(w)\leftarrow {\hat{\lambda }}^{n}(w)+\tau \left(\hat{f}(w)-\sum_{k}{\hat{u}}_{k}^{n+1}(w)\right)$$

where $w_{k}$ represents the center frequency of each mode, $\hat{f}(w)$ signifies the Fourier spectrum of the original signal, τ is the parameter of noise tolerance.

The algorithm iterates until the convergence criteria are met or the maximum number of iterations is reached. Convergence is deemed achieved when the following conditions are satisfied:

(8) $$\frac{{\sum_{k}\left\|{\hat{u}}_{k}^{n+1}-{\hat{u}}_{k}^{n}\right\|}_{2}^{2}}{{\left\|{\hat{u}}_{k}^{n}\right\|}_{2}^{2}}<\varepsilon$$

where ${\hat{u}}_{k}$ denotes the Fourier spectrum of the *k*-th mode, ε denotes the convergence criterion tolerance (generally set to $10^{-6}$), n indicates the iteration count.

### 2.2. Walrus Optimizer (WO)

The WO is a swarm-intelligence-based optimization method. It achieves efficient search by simulating walruses’ social structure and behavioral strategies, including population modeling and responses to ‘danger’ and ‘safety’ signals. The social hierarchy among adult, juvenile, and female individuals is also considered. These mechanisms collectively balance global exploration and exploitation to enhance search efficiency. This section provides an overview of the main concepts and core mechanisms of the WO algorithm.

The optimization process of WO begins with the generation of an initial population of candidate solutions, which are randomly initialized within the predefined upper and lower bounds of the design variables. The diversity of the population facilitates comprehensive coverage of the search space, thereby increasing the probability of discovering potential global optima. During iterations, individuals update their positions based on social interactions and environmental signals. Over time, the population gradually converges toward the best solution found so far.

The WO algorithm simulates the response of walruses to environmental changes by introducing ‘safety’ and ‘danger’ signals. These two signals jointly influence individual behaviors, guiding the population toward promising regions of the search space. The danger signal represents the risk level at an individual’s current position, as calculated in Equation (9) [24]. As the optimization progresses, this risk signal gradually diminishes, promoting convergence toward feasible regions. In contrast, the safety signal, computed according to Equation (13), is used to indicate the attractiveness of an individual’s current position. Its intensity increases progressively over iterations, thereby enhancing local exploitation ability. By effectively balancing exploration and exploitation under the ‘safety–danger’ mechanism, WO enables efficient navigation of the search space. Note that α denotes the VMD penalty factor in this paper; to avoid confusion, the coefficient α in the original WO formulation is denoted as γ hereafter.

(9) Danger_signal=A×R

(10) A=2×γ

(11) $$\gamma =1-\frac{l}{I}$$

(12) $$R=2\times r_{1}-1$$

(13) $$Safety\text{\_}signal=r_{2}$$

where *A* and *R* are danger factors; γ gradually decreases from 1 to 0 with iteration. The safety signal is denoted as $r_{2}$, and the random variables $r_{1}$ and $r_{2}$ are random numbers that lie in the range of (0, 1). The variable l represents the current iteration index, whereas I denotes the maximum number of iterations.

The migration phase is designed to enhance the global exploration capability of the algorithm. During this phase, walrus individuals update their positions toward unexplored regions of the search space, with random variables and migration step sizes controlling the update process (see Equations (14)–(16)).

(14) $$X_{ij}^{l+1}=X_{ij}^{l}+Migration\text{\_}step$$

(15) $$Migration\text{\_}step=(X_{m}^{l}-X_{n}^{l})\times \beta \times r_{3}^{2}$$

(16) $$\beta =1-\frac{1}{1+e^{\frac{-10(l-0.5I)}{I}}}$$

where the modified position in iteration *i* along dimension *j* is represented by $X_{ij}^{l+1}$, two randomly selected positions are indicated by $X_{m}^{l}$ and $X_{n}^{l}$, β is the migration step control factor, which varies with iteration as a smooth curve, and $r_{3}$ is a random number that lies in the range of (0, 1).

The reproduction phase promotes population diversification, with male, female, and juvenile individuals evolving according to their respective behavioral mechanisms, while random variables and iteration-dependent parameters further influence their trajectories. Male walruses act as scouts, exploring the search space and updating their positions accordingly. Female walruses focus on exploitation, refining potential solutions in promising regions. Juveniles interact with both males and females, enhancing diversity by combining exploratory and exploitative behaviors. Random perturbations are applied to their position updates to further improve exploratory capability. Through these complementary behaviors, WO balances exploration and exploitation, facilitating convergence toward optimal solutions. The mathematical formulations governing this stage are provided in Equations (17)–(19) [24].

(17) $$female_{ij}^{l+1}=female_{ij}^{l}+\gamma \times (male_{ij}^{l}-female_{ij}^{l})+(1-\gamma )\times (X_{best}^{l}-female_{ij}^{l})$$

(18) $$Juvenile_{ij}^{l+1}=(O-Juvenile_{ij}^{l})\times P$$

(19) $$O=X_{best}^{t}+Juvenile_{ij}^{t}\times LF$$

where P represents the distress coefficient of juvenile walruses and is a random number of (0, 1), O represents the safety position, LF is a vector of random numbers based on L’evy distribution representing L’evy movement [24].

### 2.3. Minimum Mode Envelope Entropy (MMEE)

To optimize the VMD parameters, we adopt minimum mode envelope entropy (MMEE) as the fitness criterion. During rapid keystroke movements, sEMG typically exhibits sustained high activation, producing envelope fluctuations that are structured and physiologically constrained, in contrast to the stochastic envelope behavior of Gaussian white noise. Envelope entropy provides an effective measure of the temporal randomness of the time-normalized envelope distribution: lower values indicate more structured, physiologically meaningful activity, whereas higher values correspond to noise-dominated modes. Accordingly, a well-parameterized VMD is expected to concentrate informative sEMG activity into at least one mode whose envelope is temporally concentrated and thus has low entropy; therefore, the fitness is defined as the minimum envelope entropy across modes. To avoid trivial low-energy modes yielding artificially low entropy, the minimization is performed only over modes whose energy exceeds a small threshold (energy gating). The MMEE computation procedure is described as follows.

First, the *k*-th mode component $u_{k}(t)$ obtained from VMD is subjected to the Hilbert transform to construct its analytic signal, from which the envelope amplitude $a_{k}(t)$ is extracted.

(20) $$a_{k}(t)=\sqrt{u_{k}^{2}(t)+{\hat{u}}_{k}^{2}(t)}$$

where ${\hat{u}}_{k}^{2}(t)$ is obtained as the Hilbert transform of $u_{k}^{2}(t)$.

Next, the envelope signal $a_{k}(t)$ is normalized to construct the probability distribution sequence $p_{k,j}$.

(21) $$p_{k,j}=\frac{a_{k}(j)}{\sum_{j=1}^{N}a_{k}(j)}$$

Here, N denotes the number of sampled points, and j=1,2,3,…,N.

The envelope entropy of the k-th mode is computed according to the definition of Shannon entropy:

(22) $$E_{k}=-\sum_{j=1}^{N}p_{k,j}\log_{2}p_{k,j}$$

Finally, the fitness function is defined as the minimum envelope entropy among all K modes. This strategy is designed to guide the optimization process toward parameter configurations that yield the most distinct and noise-suppressed mode components:

(23) $$Fitness=\min\left\{E_{1},E_{2},\ldots ,E_{K}\right\}$$

During the optimization process, a smaller fitness value indicates a more desirable VMD performance and a higher degree of separation between the useful signal and noise.

### 2.4. WO-VMD

As a non-recursive signal decomposition technique, VMD has shown strong performance in processing nonlinear and nonstationary sEMG signals. However, its decomposition performance strongly depends on the preset parameters, namely the number of modes *K* and the penalty factor α. A small value of *K* may lead to mode mixing and loss of useful information, whereas an excessively large *K* may cause over-decomposition and produce spurious components. The penalty factor determines the bandwidth of each mode and directly influences the effectiveness of noise suppression.

To overcome the subjectivity of manually selecting parameters, VMD was integrated with the WO to form the WO-VMD method. The method leverages the strong global search capability of WO and uses the MMEE as the fitness function to automatically search the parameter space and achieve optimal noise reduction. The algorithmic procedure of WO-VMD is summarized as follows.

Step 1: Initialization. The population size *N* and maximum number of iterations I are specified. The bounds of the optimization variables are defined: *K* is searched within $\left[K_{\min},K_{\max}\right]$ (rounded to integers), and α within $\left[\alpha_{\min},\alpha_{\max}\right]$. The initial population positions $X_{i}=[K_{i},\alpha_{i}](i=1,2,\ldots N)$. In this study, the search bounds were fixed to *K* = 2–15 and *α* = 500–2000.

Steps 2–5: Main loop (for l=1,2,…,I).

Step 2: Fitness evaluation. For each individual $X_{i}$, the noisy signal is decomposed using VMD with its parameter pair $K_{i},\alpha_{i}$. The envelope entropy of each mode is computed according to Equations (20)–(23), and the minimum entropy is taken as the fitness $F_{i}$.

Step 3: Update of the global optimum. The fitness values of all individuals are compared, and the global best position $X_{best}$ and its fitness $F_{best}$ are updated.

Step 4: Position update. Population positions are updated according to the WO update rules driven by *danger* and *safety* signals, which govern the switching between exploration and exploitation

Step 5: Boundary check and termination. Updated positions are checked against the boundaries, and corrections are applied if needed. If l<I, the iteration continues; otherwise, the global optimal parameters $K_{opt}$, $\alpha_{opt}$ are output.

Step 6: Optimal decomposition. The optimal parameters $K_{opt},\alpha_{opt}$ are used to perform the final VMD of the original signal.

Step 7: Mode screening. The Hilbert transform is applied to each mode, and suitable components are selected for signal reconstruction.

**Figure 2:**
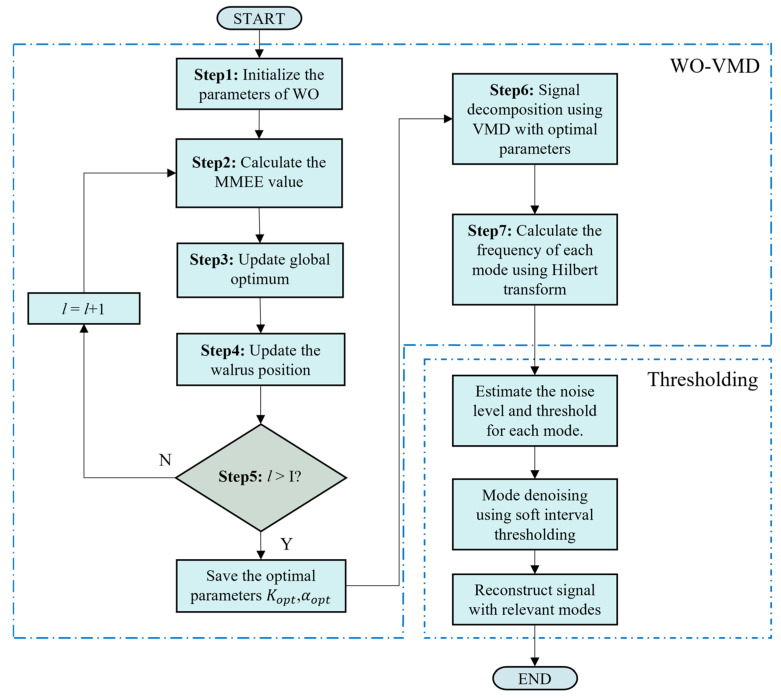
Flowchart of WO-VMD with Thresholding.

The overall framework is shown in Figure 2, where the thresholding strategy is further detailed in Section 2.5

### 2.5. Thresholding Strategy and Improved Threshold Estimation

Conventional pointwise thresholding often introduces discontinuities at threshold boundaries, which may produce Gibbs-type ringing in the reconstructed signal. To address this issue, interval-based thresholding divides the waveform into continuous segments based on its zero-crossings and applies thresholding within each segment, thereby avoiding the discontinuities introduced by direct pointwise thresholding. Each mode can be partitioned into multiple intervals based on its zero crossings. Within each interval $Z_{i}^{\left(j\right)}$, the hard and the soft interval thresholding can be expressed, respectively, as [15]:

(24) $${\tilde{u}}_{i}(Z_{i}^{\left(j\right)})=\left\{\begin{array}{cc} u_{i}(Z_{i}^{\left(j\right)}) & \left|u_{i}(q_{i}^{\left(j\right)})\right|>T_{i}\\ 0 & \left|u_{i}(q_{i}^{\left(j\right)})\right|\leq T_{i} \end{array}\right.$$

and

(25) $${\tilde{u}}_{i}(Z_{i}^{\left(j\right)})=\left\{\begin{array}{cc} u_{i}(Z_{i}^{\left(j\right)})\frac{\left|u_{i}(q_{i}^{\left(j\right)})\right|-T_{i}}{u_{i}(q_{i}^{\left(j\right)})} & \left|u_{i}(q_{i}^{\left(j\right)})\right|>T_{i}\\ 0 & \left|u_{i}(q_{i}^{\left(j\right)})\right|\leq T_{i} \end{array}\right.$$

where $Z_{i}^{\left(j\right)}=[z_{i}^{\left(j\right)}z_{i}^{\left(j+1\right)}]$,$z_{i}^{\left(j\right)}$ is the *j*-th adjacent zero-crossing of the *i*-th mode, $u_{i}(q_{i}^{\left(j\right)})$ represents the single extreme of the adjacent zero-crossing interval $Z_{i}^{\left(j\right)}$, $u_{i}(Z_{i}^{\left(j\right)})$ and ${\tilde{u}}_{i}(Z_{i}^{\left(j\right)})$ represent the raw signal and the corresponding processed signal.

The threshold $T_{i}$ is typically computed using the universal threshold formula, $T_{univ}=\sigma \sqrt{2\ln N}$, where *N* is the total signal length. An overly large threshold over-attenuates informative components, whereas an overly small threshold fails to provide sufficient noise suppression. For sEMG recorded during rapid keystroke actions, muscle activation is high and sustained. Informative sEMG components are therefore prone to thresholding-induced over-attenuation, especially when shrinkage-based operators (e.g., soft thresholding) are used, which may reduce reconstruction fidelity. An improved adaptive threshold $T_{i}$ is proposed to mitigate these issues. Noise statistics are characterized using the length of a local resting segment, $N_{rest}$. In our experiments, $N_{rest}$ corresponds to the first 5 s relaxed period in each trial and is automatically determined from the protocol timing. An operator-dependent calibration coefficient C is introduced to accommodate the shrinkage behavior of different thresholding operators. The threshold is defined as:

(26) $$T_{i}=C\sigma_{i}\sqrt{2\ln N_{rest}}$$

where $N_{rest}$ represents the number of samples in the resting period. $\sigma_{i}$ denotes the noise standard deviation. It is estimated using a robust median-based estimator computed from signal components. In this setting, high-duty-cycle muscle activity inflates the global noise estimate. Noise-level overestimation is avoided by estimating the noise variance from the median of resting segments. In this study, the resting segment corresponds to the 5 s pre-task relaxed interval recorded at the beginning of each trial (Section 3.1). The formula is given below:

(27) $$\sigma_{i}=\frac{median(\left|u_{i}^{rest}(t)\right|)}{0.6745}$$

where $u_{i}^{rest}(t)$ represents the resting segments of $u_{i}(t)$.

According to the findings reported by Kopsinis et al. [15], the selection of the coefficient *C* is critical and depends on the specific characteristics of the thresholding operator. For the hard thresholding operator, which retains the original amplitude of supra-threshold components, the signal energy is relatively insensitive to the threshold level. Therefore, a larger coefficient (e.g., $C\in \left[0.6,0.8\right]$) is preferred to strengthen noise suppression without attenuating the peak intensity of valid muscle activities. In contrast, the soft thresholding typically shrinks the amplitude of the retained mode samples, which may reduce signal energy and attenuate informative myoelectric activity. To prevent physiologically meaningful activity from being excessively attenuated, a smaller coefficient (e.g., $C\in \left[0.3,0.4\right]$) is adopted. This configuration balances the trade-off between effective noise removal and the preservation of signal fidelity. In the proposed pipeline, soft interval thresholding is used with C=0.3; hard thresholding is only used for comparison in Section 3.3.

### 2.6. Performance Evaluation

To evaluate the performance of denoising methods, the SNR and RMSE are commonly used as evaluation metrics [31]. For each trial, the recorded sEMG segment before artificial WGN addition was treated as the reference signal, and the denoised output was evaluated against this reference. The formulas for these metrics are given below:

(28) $$SNR=10\log_{10}(\frac{\sum_{n=1}^{N}x{\left(n\right)}^{2}}{\sum_{n=1}^{N}{\left[x(n)-\hat{x}(n)\right]}^{2}})$$

(29) $$RMSE=\sqrt{\frac{1}{N}\sum_{n=1}^{N}{\left[x(n)-\hat{x}(n)\right]}^{2}}$$

where x(n) is the reference signal, $\hat{x}(n)$ is the reconstructed signal, and *N* is the signal length.

In this study, the denoising performance is quantified using the ΔSNR and the ΔRMSE%. In addition, to evaluate the fidelity of physiological features after denoising, the ΔMDF%, where MDF denotes the median frequency, is further introduced as a supplementary metric. The calculation formulas of the above metrics are as follows:

(30) $$\Delta SNR=SNR_{Output}-SNR_{Input}$$

Here, $SNR_{Output}$ and $SNR_{Input}$ denote the SNR values before and after denoising, respectively.

(31) $$\Delta RMSE\%=\frac{RMSE_{noisy}-RMSE_{denoised}}{RMSE_{noisy}}\times 100\%$$

Here, $RMSE_{noisy}$ and $RMSE_{denoised}$ denote the RMSE of the noisy signal and the denoised signal relative to the reference signal, respectively.

The definition of MDF satisfies the following equation:

(32) $$\int_{0}^{MDF}P(f)df=\frac{1}{2}\int_{0}^{f_{s}/2}P(f)df$$

Here, P(f) denotes the power spectral density of the signal, and $f_{s}$ is the sampling frequency.

(33) $$\Delta MDF\%=\frac{\left|MDF_{processed}-MDF_{reference}\right|}{MDF_{reference}}\times 100\%$$

Here, $MDF_{processed}$ and $MDF_{reference}$ denote the MDF of the denoised signal and the reference signal.

## 3. Experiments and Results

### 3.1. Participants and Procedure

Eighteen healthy volunteers (13 men and 5 women; aged 19–25 years) were recruited for this study. All participants were screened and confirmed to be free from cardiovascular, neuromuscular, and metabolic disorders. Prior to the experiment, participants were fully informed of the study objectives, procedures, and potential risks, and written informed consent was obtained. Participants refrained from vigorous exercise and abstained from caffeine, nicotine, and alcohol for at least 48 h before testing. Experimental procedures are conducted in accordance with the Declaration of Helsinki, and the study protocol was approved by the local Ethics Committee of Tianjin University.

**Figure 3:**
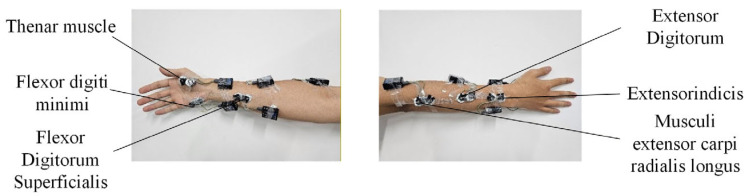
Position of the sEMG electrodes on the forearm.

Before electrode placement, the skin surface was cleaned with alcohol to remove the stratum corneum and reduce skin impedance, thereby minimizing the influence of motion artifacts during signal acquisition [32]. The experimental task in this study involves finger keystroke actions, and the muscles were selected from the perspective of their functional relevance to finger motor control during the keystroke process. sEMG sensors were then placed on the abductor pollicis brevis (APB), flexor digiti minimi (FDM), flexor digitorum superficialis (FDS), extensor digitorum (ED), extensor indicis (EI), and extensor carpi radialis longus (ECRL) according to the SENIAM guidelines [33]. The sensors were manufactured by Noraxon, and the signals were sampled at a rate of 1500 Hz. Specific sensor locations on the right forearm are shown in Figure 3. To ensure sensor stability during the task, the electrodes and leads were secured with athletic tape.

**Figure 4:**
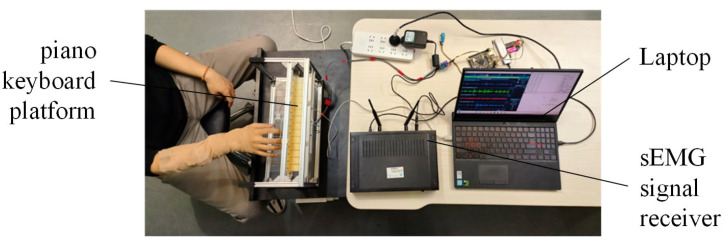
The experimental setup.

During the experiment, a continuous keystroke task was performed on a mock piano-keyboard platform at 60 beats per minute (BPM). At the beginning of each trial, participants were instructed to remain relaxed for 5 s before performing the keystroke task. This pre-task interval was recorded as a resting segment and was later used for noise-level estimation in the proposed thresholding scheme. Each participant completed ten trials, with 20 keystrokes per trial. Adequate rest was provided between trials to reduce fatigue-related effects on signal quality and task performance. The experimental setup is shown in Figure 4. In the subsequent sections, the denoising performance of the proposed method is analyzed and compared using the sEMG signals recorded from all monitored muscles in the experiment.

### 3.2. Thresholding Method Analysis

The thresholding stage serves as a key component, and its performance directly affects signal fidelity and noise suppression. The sEMG signals examined in this study were recorded during rapid keystroke tasks and exhibit prominent, brief burst-like activity and a high proportion of active segments, which limits the applicability of universal threshold methods.

The factors that influence thresholding performance were systematically analyzed from two perspectives. First, the type of thresholding operator was examined. Different operators have distinct mathematical properties and amplitude-shrinkage behaviors, which can affect denoising results. Second, the threshold estimation formula was investigated. The threshold estimation formula directly determines the effective strength and operating range of the operator, thereby affecting overall thresholding performance. A systematic comparison and discussion of these factors help clarify how thresholding strategies differ across noise levels and signal characteristics and provide theoretical support for further improvements in denoising algorithms.

#### 3.2.1. Comparison of Thresholding Operators

Two thresholding operators are commonly used: hard thresholding and soft thresholding. Hard thresholding better preserves the original amplitudes of valid components; however, continuity and smoothness near the threshold boundary are limited. Soft thresholding shrinks coefficients above the threshold, yielding a smoother transition and mitigating discontinuity artifacts during reconstruction. Denoising performance was compared between hard and soft thresholding under different noise levels using both the universal threshold and the proposed improved threshold.

**Figure 5:**
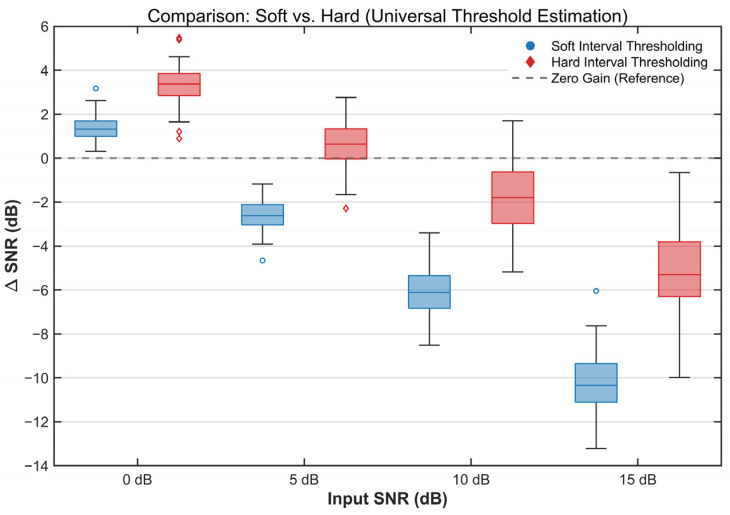
Denoising performance using the universal threshold estimation.

The denoising results obtained by applying hard and soft thresholding to the sEMG signals using the universal threshold are shown in Figure 5. Contrary to typical reports in prior studies, the denoising performance of soft thresholding was lower than that of hard thresholding across the tested noise levels. This pattern can be attributed to the high-activation and high-duty-cycle sEMG signals analyzed in this study. The universal threshold, which relies on a median-based noise estimate, tends to overestimate the threshold value under these conditions. Soft thresholding continuously shrinks amplitude above the threshold, which can over-attenuate informative sEMG components and thereby degrade reconstruction quality and denoising performance. In contrast, hard thresholding retains coefficients above the threshold and suppresses only those below it; thus, it is less sensitive to threshold overestimation.

Table 1 reports the evaluation results of the two thresholding operators under the improved threshold estimation formula across different input SNRs. The results indicate that both operators achieved higher output SNR than the universal threshold. The improved estimator provided a larger gain for soft thresholding, and soft thresholding outperformed hard thresholding in denoising performance under this setting.

#### 3.2.2. Comparison of Threshold Estimation Formulas

A controlled experiment was designed to evaluate the impact of different threshold estimation schemes on sEMG denoising performance. All experiments employed the proposed WO-VMD method. Four threshold estimation schemes were considered: T1 used a zero-threshold baseline; T2 applied the universal threshold; T3 estimated the noise level from resting segments; and T4 introduced a coefficient C based on T3. These four strategies were compared under various input noise levels using soft interval thresholding.

**Figure 6:**
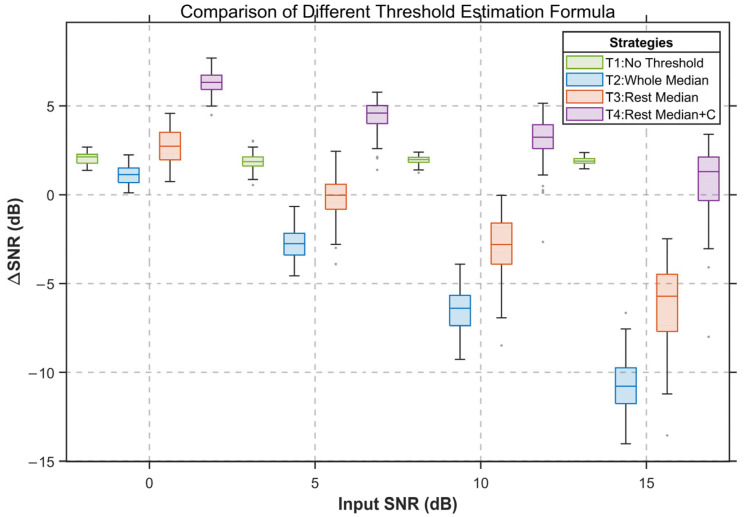
The influence of different threshold estimation formulas on denoising performance.

The denoising results are shown in Figure 6, where T1, T2, T3, and T4 are marked in green, blue, red, and purple, respectively. The figures show that the standard universal threshold (T2) yields the lowest ΔSNR across all noise conditions. This occurs because prolonged muscle activation during rapid keystroke tasks prevents the global median from accurately reflecting the true noise level, leading to threshold overestimation; moreover, soft thresholding shrinks the amplitudes of suprathreshold components, suppressing valid sEMG activity and degrading denoising performance.

With noise estimated from resting segments in T3, the overestimation issue is substantially alleviated, and its denoising performance surpasses that of the universal threshold scheme across all noise levels. However, because soft thresholding inherently applies amplitude shrinkage, meaningful amplitude sEMG components may still be partially suppressed, thereby limiting the performance improvement of T3, particularly under high-SNR conditions.

In T4, introducing the coefficient C reduces the threshold magnitude and effectively mitigates the excessive shrinkage induced by soft thresholding, allowing valid sEMG components to be preserved to a greater extent. Experimental results show that T4 achieves a better performance, with particularly strong improvements at low input SNR. For example, when the input SNR is 5 dB, T4 improves the ΔSNR by approximately 7 dB relative to T2, demonstrating the advantage of the proposed thresholding strategy under severe noise conditions.

### 3.3. The Application of the Proposed Method

**Figure 7:**
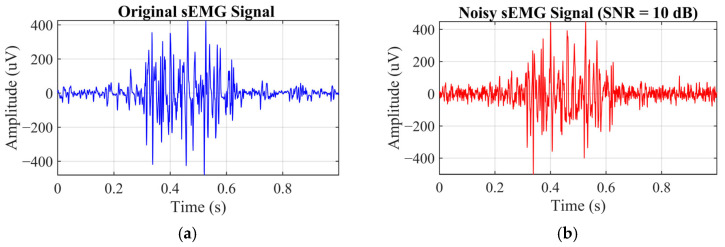
An example of the collected signals. (**a**) Original sEMG signals. (**b**) Noisy sEMG signals (input SNR = 10 dB).

**Figure 8:**
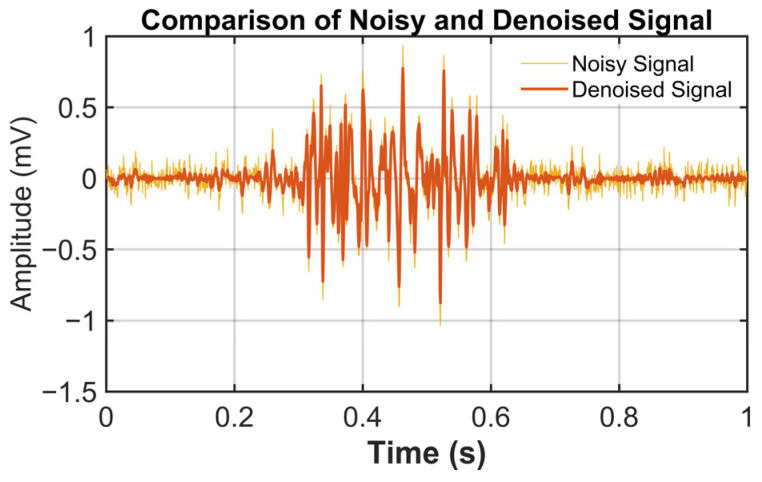
Denoising results using proposed method (input SNR = 10 dB).

Denoising performance was systematically evaluated by adding noise to the signals at four input SNR levels: 0, 5, 10, and 15 dB. Figure 7 presents an example sEMG segment from a single index-finger keystroke, including the original signal and the signal contaminated at an input SNR of 10 dB. Figure 8 shows the denoising result for a segment at the input SNR of 10 dB. As shown in the figure, additive white Gaussian noise was effectively attenuated, while physiologically meaningful muscle-activation components were well preserved. Denoising results for this sEMG segment at other input SNR levels are summarized in Table 2. As shown in Figure 8 and Table 2, key sEMG features are well recovered from noisy observations by the proposed method. Effective noise suppression is achieved while high signal fidelity is maintained, indicating the robustness of the proposed denoising scheme.

### 3.4. The Comparison of Denoising Performance

**Figure 9:**
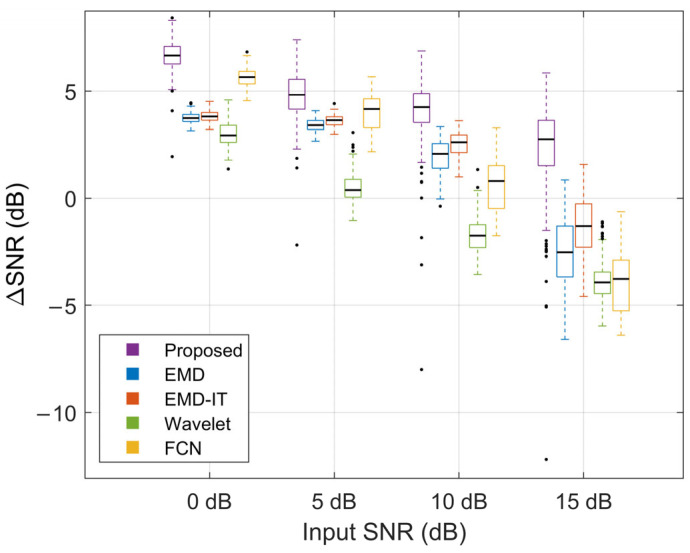
Performance evaluation using ΔSNR.

**Figure 10:**
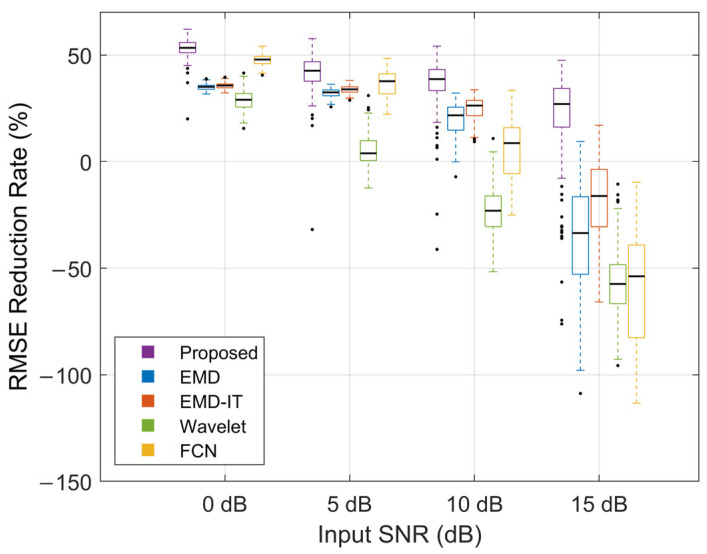
Performance evaluation using ΔRMSE%.

**Figure 11:**
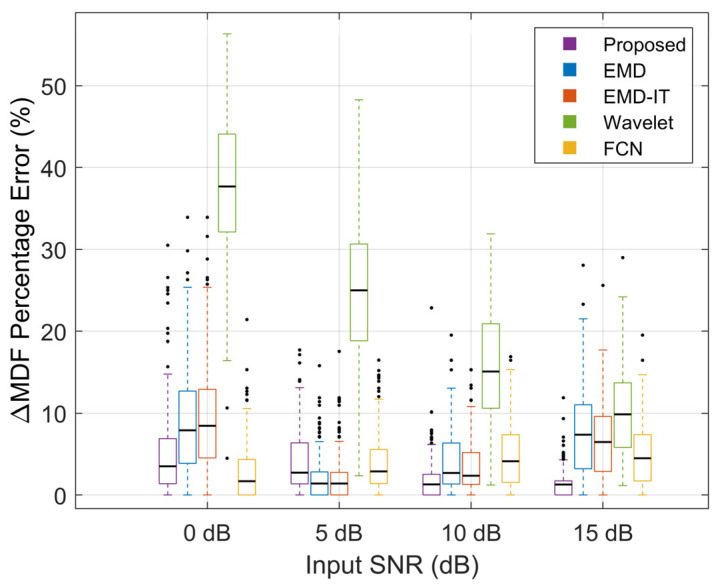
Performance evaluation using ΔMDF%.

Figure 9, Figure 10 and Figure 11 illustrate the denoising performance of different methods. This comparison evaluates the proposed method alongside wavelet denoising, EMD-based denoising, EMD-based indirect thresholding (EMD-IT) and an FCN-based denoising autoencoder under different input SNR levels. In the comparative experiments in this section, EMD-based denoising methods were implemented with the same threshold estimation scheme and operator to minimize the impact of differences in thresholding strategy on the comparisons.

As shown by the overall trends in Figure 9 and Figure 10, the metric medians decreased as the input SNR increased, indicating less room for improvement under light-noise conditions. Across all noise levels, the proposed method achieved higher ΔSNR and ΔRMSE% than the other methods. Specifically, at input SNR levels of 0, 5, 10, and 15 dB, the proposed method achieved median ΔSNR values of 6.65, 4.82, 4.24, and 2.75 dB and median ΔRMSE% values of 53.38%, 42.62%, 38.68%, and 27.03%, respectively. At 15 dB, the distributions of both metrics remained positive, indicating a net gain even at a high input SNR. Under heavy-noise conditions, the proposed method exhibited more compact boxplots, suggesting more stable improvements and robust performance. Under light-noise conditions, more extreme outliers were observed for the proposed method. Such outliers may reflect occasional failures caused by over-attenuation at a small number of boundary samples. In addition, a smaller denominator in the RMSE-reduction ratio under this condition can amplify outliers, leading to more pronounced extreme values. At input SNRs of 0 dB and 5 dB, FCN outperformed EMD and EMD-IT. The opposite trend was observed at 10 dB and 15 dB, suggesting that FCN is better suited to heavy-noise scenarios. At 15 dB, all baseline methods exhibited marked performance degradation, suggesting that informative sEMG components may have been misclassified as noise and attenuated.

Figure 11 indicates that the proposed method consistently yields low ΔMDF% errors. At input SNRs of 10 dB and 15 dB, the error remains low with a relatively stable distribution, indicating that effective denoising is achieved while the spectral structure of the signal is well preserved. Low ΔMDF% errors were also observed for FCN across noise levels, indicating good fidelity of physiological features after denoising. For the wavelet-based method, ΔMDF% was higher and the interquartile range (IQR) was wider at input SNRs of 0–10 dB, suggesting the largest perturbation of MDF and poor stability. In contrast, lower MDF errors were observed for EMD and EMD-IT at moderate noise levels. At 15 dB, both the error magnitude and dispersion increased, suggesting that the reconstruction process is more likely to induce spectral shifts under light-noise conditions.

Considering ΔRMSE%, ΔSNR, and ΔMDF% together, stable error reductions and SNR gains were achieved by the proposed method across the full range of 0–15 dB, with low errors maintained in MDF preservation. In contrast, the wavelet method exhibited marked MDF shifts and greater dispersion under multiple noise conditions. EMD/EMD-IT and FCN showed clear degradation in ΔSNR and ΔRMSE% at a high SNR of 15 dB, suggesting a higher risk of over-processing in light-noise settings. These results indicate that the proposed method achieves a more favorable adaptive trade-off between noise reduction and feature fidelity, while improving robustness.

Table 3 summarizes the statistical analysis of these results. The Friedman test was applied under each input condition. Significant overall differences were observed among methods for all three metrics across noise levels (*p* < 0.05). Post hoc pairwise comparisons were performed using the Wilcoxon signed-rank test, with Holm–Bonferroni correction. Comparisons between the proposed method and each baseline were emphasized. For denoising gain, significantly higher ΔSNR and significantly larger ΔRMSE% were obtained with the proposed method under all input SNR conditions, outperforming all baselines. For physiological feature preservation, ΔMDF% differed significantly from those of the traditional baselines across noise levels. The only exception occurred at 5 dB, where no significant difference in ΔMDF% was found between the proposed method and FCN (*p* = 0.85).

Runtime is an important metric for evaluating an algorithm’s performance. We applied five methods to denoise several 1 s signal segments, and the results are summarized in Table 4. It reports the average runtime together with the median values of the three metrics at an input SNR of 15 dB. As shown in the table, the proposed method achieves a clear advantage in denoising performance, whereas the other methods tend to cause over-smoothing of the signal, which also comes at the cost of a longer runtime for the proposed approach.

## 4. Discussion

Within the established VMD framework, the WO was integrated, and the MMEE was adopted as the fitness metric. Key parameters *K* and α were globally optimized in an adaptive manner, thereby reducing uncertainty introduced by empirical tuning. Compared with conventional empirical settings, decomposition stability under different noise conditions is improved, and the separability between informative and noise components is enhanced. Experimental results demonstrate improved performance across noise levels, with consistent advantages over multiple baseline methods. Overall, MMEE is highly sensitive to noise variations and is well-suited for the proposed framework. It provides a clear and noise-aware criterion for optimizing parameters in decomposition-based denoising.

During thresholding, the universal threshold strategy was adapted to rapid keystroke tasks characterized by long-lasting, high-duty-cycle muscle activation. Unlike conventional threshold estimation using statistics from the whole segment, we focused on characterizing noise using resting segments and explicitly considered differences between operators. Thus, over-suppression of informative components caused by threshold overestimation is reduced. The results support an improved balance between noise suppression and signal fidelity. Physiologically meaningful sEMG activity is preserved while noise is attenuated. These findings suggest that threshold estimation for nonstationary biosignals should be tailored to match noise characteristics with signal structure. Otherwise, biased noise estimation can lead to excessive attenuation of informative components.

The combined WO-VMD and improved threshold estimation strategy also exhibited good structural transferability. This behavior is largely driven by accurate estimation of noise level and adaptive adjustment of the decomposition scale. The result suggests that the proposed strategy may be beneficial for other nonstationary biomedical signals and provide useful guidance for related signal-processing tasks. Moreover, the threshold formula is designed to be customized for specific signal types, enabling a flexible denoising approach across application domains.

Despite the strong performance of the proposed method, the study has several limitations. The experimental data were collected mainly from younger subjects. Given that the spectral characteristics and noise properties of sEMG signals may vary with age and across different neuromuscular disorders, applying the method to older adults or patients with neuromuscular diseases may require adjusting the noise-estimation level and threshold parameters according to the actual signal characteristics. Therefore, future work should include participants across a wider age range and diverse health conditions to adequately assess population-level applicability. Furthermore, the parameter-search process inherent to WO-VMD introduces a notable computational cost. This computational burden currently limits the practical deployment of the method in real-time settings. As shown in Table 4, the proposed method requires iterative computations during parameter optimization, resulting in substantial runtime that increases approximately proportionally with the number of fitness-function evaluations in the optimizer. If deployed on embedded gesture-recognition devices, feasibility could be improved by using less demanding parameter settings and/or performing offline optimization when appropriate. Overall, the proposed method is more suitable for applications with less stringent real-time requirements or for offline use to obtain high-quality sEMG signals. Reducing this computational complexity while maintaining denoising performance will remain an important direction for future research. Finally, threshold estimation currently depends on noise characteristics observed during resting periods. Consequently, signals that lack clear inactive periods may require more robust estimation strategies. For datasets without an explicitly designed resting segment, a sliding-window-based resting-segment detection approach can be used, for example, by identifying sustained low-activity windows based on measures such as root mean square (RMS) or signal energy to automatically extract resting segments for noise-level estimation.

Overall, the study introduces improvements in two key areas: parameter adaptivity and the accuracy of noise estimation. Accordingly, it offers a robust and generalizable framework for high-quality reconstruction of sEMG signals.

## 5. Conclusions

A denoising method based on WO-VMD and improved threshold estimation is proposed for sEMG signals recorded during rapid keystroke tasks. Two key issues are addressed in existing WO-VMD-based denoising method. First, key parameters in VMD, such as the number of modes *K* and the penalty factor α, are typically set empirically. Second, during rapid keystroke actions, universal threshold estimation formulas often overestimate the threshold, and informative components can be excessively attenuated by soft thresholding. The experimental results for both ΔSNR and ΔRMSE% indicate that the proposed method outperforms representative benchmark approaches in denoising performance across all tested noise levels. The ΔMDF% results further demonstrate that the proposed method also achieves favorable performance in preserving physiological signal features. Overall, the proposed method provides a robust and practical solution for reliable sEMG analysis during rapid keystroke tasks. In addition, the proposed strategies for adaptive parameter optimization and improved threshold estimation may also be applicable to other biosignal scenarios in which prolonged informative activity can bias conventional noise estimation and lead to over-attenuation, such as sustained high-duty-cycle EMG tasks (e.g., tremor-like repetitive contractions and typing-like industrial operations).

## Figures and Tables

**Table 1:** Results of ΔSNR under the improved threshold estimation.

Input SNR	0 dB	5 dB	10 dB	15 dB
**Soft Thresholding**	6.6526	4.8160	4.2494	2.7514
**Hard Thresholding**	5.4205	3.1719	1.5420	−1.5628

**Table 2:** Denoising performance of proposed method under different input SNRs.

Input SNR	0 dB	5 dB	10 dB	15 dB
**Output SNR**	6.7602	9.9007	12.8533	15.8779
**RMSE**	0.0805	0.0566	0.0496	0.0439

**Table 3:** Statistical analysis of the denoising performance.

Metric	Noise Level	Friedman (*p*)	Proposed & Other Baseline (Adj.p)
**Δ** **SNR**	0, 5, 10, 15 dB	<0.05	<0.05
**Δ** **RMSE%**	0, 5, 10, 15 dB	<0.05	<0.05
**Δ** **MDF%**	0, 5, 10, 15 dB	<0.05	<0.05 5 dB vs. FCN (0.85)

**Table 4:** A comparison of execution time.

Method	Wavelet	EMD	EMD-IT	FCN	Proposed Method
**Time (s)**	0.006	0.013	0.018	0.008	1.16
**Δ** **SNR (15 dB)**	−3.93	−2.53	−1.31	−3.76	4.25
**Δ** **RMSE% (15 dB)**	−57.41	−33.49	−16.13	−53.81	27.03
**Δ** **MDF% (15 dB)**	9.85	28.04	25.60	4.47	1.28

## Data Availability

The data cannot be made public due to ethical reasons.
